# Surgically repaired tetralogy of Fallot in the 7th decade: a late presentation of severe pulmonic regurgitation

**DOI:** 10.1186/s43044-024-00477-3

**Published:** 2024-04-14

**Authors:** Kyle Varkoly, Akarsh Parekh, Melissa Ianitelli, Mostafa Hamada, Alexandra Lucas, Thomas Forbes

**Affiliations:** 1 Department of Internal Medicine, McLaren Macomb Hospital- Michigan State University College of Human Medicine, Mt Clemens, MI USA; 2 Department of Cardiovascular Medicine, McLaren Macomb Hospital, Mt Clemens, MI USA; 3 College of Medicine, Kansas City University, 1750 Independence Ave, Kansas City, MO 64106 USA; 4 Center for Personalized Diagnostics, Biodesign Institute, Arizona State University, 727 E Tyler St., Tempe, AZ 85287 USA; 5 Michigan State University, East Lansing, MI USA; 6 Children’s Hospital of Michigan, Detroit Medical Center, Detroit, MI USA

**Keywords:** Tetralogy of Fallot, Surgical repair, Seventh decade, Adult congenital heart disease, Pulmonic regurgitation, Heart failure, Guidelines

## Abstract

**Background:**

Surgically repaired tetralogy of Fallot (TOF) is a congenital heart disease with a cumulative survival rate of 72% in the 4th decade of life in longitudinal single-cohort studies. Debate surrounds conservative versus surgical management in adults with TOF once pulmonary regurgitation occurs.

**Case presentation:**

A 73-year-old male with surgically corrected TOF presented with heart failure symptoms. He underwent ToF repair with a classic right Blalock–Taussig shunt at 2 years of age with transannular patching at 18 years of age. Echocardiography revealed elevated right ventricular systolic pressures, severe right ventricular dilatation, and pulmonary regurgitation. Our patient’s new-onset right-sided heart failure was managed medically with diuresis. He received a new pulmonic valve via percutaneous approach on a later planned hospitalization with resolution of symptoms and improved tricuspid regurgitation.

**Conclusion:**

It is a class I recommendation for pulmonic valve intervention once greater than moderate PR occurs; however, medical optimization should take place first. Following adequate RV load optimization, our patient underwent successful transcatheter pulmonic valve implantation with resolution of symptoms and cessation of diuretic.

## Background

Tetralogy of Fallot (TOF) is one of the most common cyanotic congenital heart diseases amendable to surgical repair. The long-term results of TOF corrective surgery and palliative shunt procedures have been promising, with a 91% 30-year survival rate [1]. Cuyper’s single-cohort longitudinal study in the Netherlands has data regarding long-term cardiovascular outcomes of TOF repair up to the 4th decade; however, data remain limited following this period, as we are just now entering our 5th decade post-TOF repair for many patients [2, 3]. Data remain limited in outcomes following this decade of life.

Delayed complications following repair of TOF include pulmonary regurgitation (PR) and right ventricular outflow tract (RVOT) aneurysms, both associated with right ventricle (RV) dilatation [4]. RVOT aneurysms are more related to transannular patching seen post-repair [4–7]. Progressive RV volume overload from longstanding PR results in chronic complications ranging from reduced exercise tolerance, reduced RV and LV systolic function, and potentially fatal atrial and ventricular tachyarrhythmias [4, 6–8]. PR has been shown to be the predominant valvular lesion prior to developing ventricular tachycardia and associated sudden death. Tricuspid regurgitation is the most common valvular lesion in patients detected prior to developing atrial fibrillation or flutter in patients with ToF [8].

Successfully managing the pulmonic regurgitation and its downstream effects is of paramount importance in this patient population. Herein we describe a case of a patient with surgically corrected TOF in the 7th decade of life and it is the first reported case in the medical literature with a successful outcome.

## Case presentation

A 73-year-old male with the past medical history of paroxysmal atrial fibrillation, essential hypertension, and surgically corrected TOF presented with worsening dyspnea and edema for 2 weeks. He had initial palliation with classic Blalock–Taussig–Thomas Shunt (BTTS) at 2 years of age rather than the aggressive surgical options available at the time, with subsequent ventricular septal defect (VSD) repair with transannular patch placement surgery at 18 years of age. He was compliant with home medications, follow-up visits, pulmonary, and cardiac rehabilitations prior to hospitalization. Home medications included Eliquis 5 mg bid, hydrochlorothiazide 25 mg qd, and metoprolol tartrate 25 mg bid. Physical examination was significant for 2 + lower extremity edema, bilateral crackles, and jugular venous distention upon arrival to the emergency department.

**Fig. 1:**
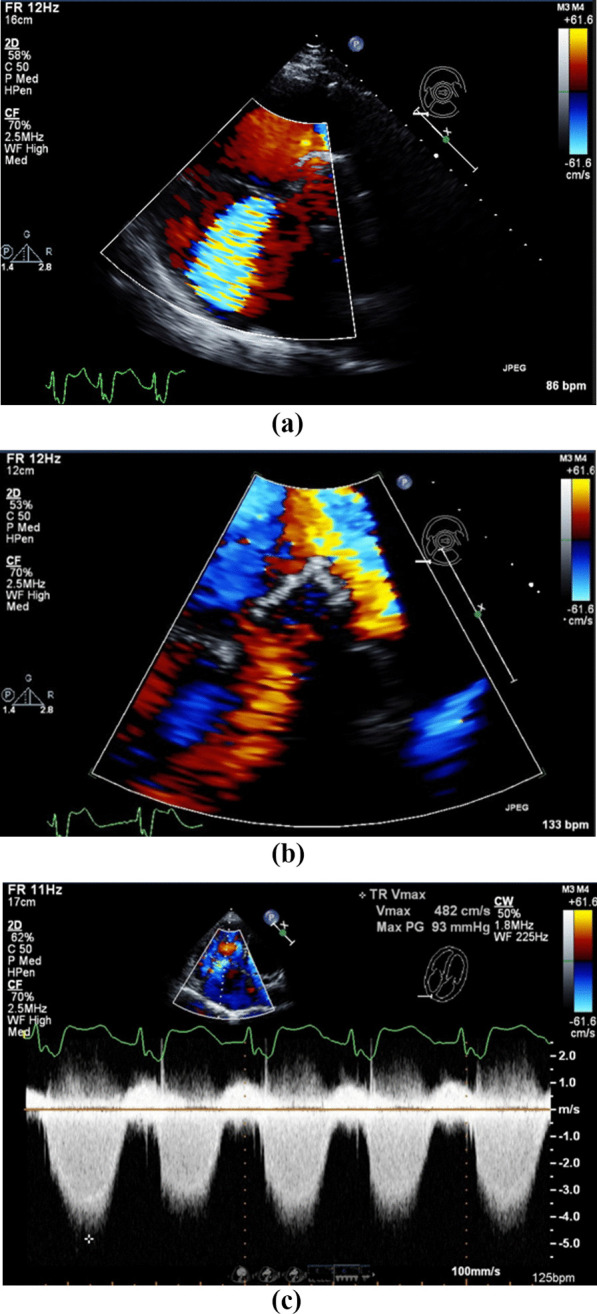
Transthoracic echocardiography revealing severe pulmonic regurgitation. **a** Parasternal short axis of the tricuspid valve inflow tract showing severe tricuspid regurgitation by color flow doppler. **b** Parasternal short-axis view with broad color flow jet covering the total diameter of the right ventricular outflow tract and showing severe pulmonic regurgitation. **c** Continuous-wave doppler through the tricuspid valve showing a peak gradient of 93 mmHg suggesting severe pulmonary hypertension in the apical view

Echocardiography (Fig. 1) revealed a preserved left ventricular ejection fraction of 60–65%, with a flattened septum in systole and diastole consistent with elevated RV pressure and volume overload. Severe RV dilatation was also seen with only a mildly reduced RV global systolic function, alone with severe PR and without pulmonic stenosis. Pulmonary artery systolic pressure was markedly elevated at 108 mmHg, estimated by TR jet velocity. Concurrently, severe tricuspid regurgitation was detected on echocardiogram.

**Fig. 2:**
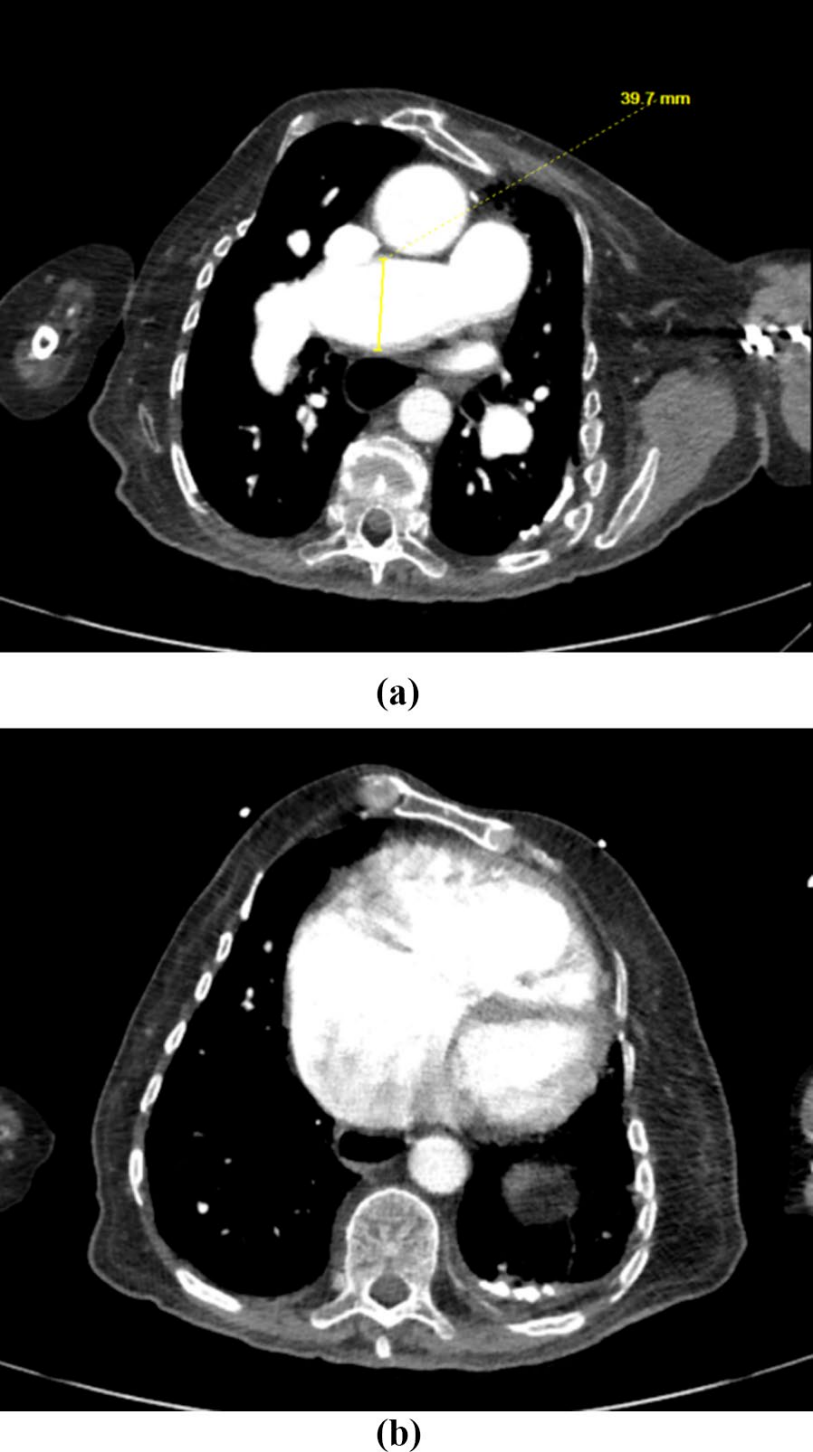
CT scan showing severe pulmonary arterial dilatation indicative of pulmonary hypertension (**a**) with severe RV dilatation (**b**)

Our patient was diuresed with IV furosemide, which was transitioned to a daily regimen of 20 mg daily per oral. The patient reported improved breathing and decreased lower extremity swelling after diuresis and was then transferred to an adult congenital heart disease center for further management of his pulmonary valve. He underwent a right and left heart catheterization on a separate staging catheterization which revealed non-obstructive epicardial coronary arteries, and he underwent cardiac computerized tomography (CT) to better delineate his pulmonary arterial anatomy (Fig. 2). Using a heart team approach, he was designated as qualified for a transcatheter valve, as he was a high-risk surgical candidate and was an excellent fit for the Harmony™ TPV 25 (Medtronic, Minneapolis, MN) pulmonic valve. Following appropriate management of his volume overload with improvement of pulmonic pressures, he was deemed ready for pulmonic intervention.

The patient ultimately underwent transcatheter pulmonary valve implantation with a Harmony™ TPV 25 (Medtronic, Minneapolis, MN). He tolerated the procedure well with resolution of HF symptoms, undergoing weekly pulmonary rehabilitation. He was discharged on his home diuretic, which was discontinued at the next office visit the following week due to concurrently improved blood pressure with resolved edema and dyspnea. Our patient’s tricuspid regurgitation resolved to trace on subsequent echocardiography.

## Conclusions

PR is present in most patients after anatomical correction of TOF, and its chronic impact on the right ventricle (RV) often manifests dramatically, as exemplified in our documented case. The literature has many reports of surgically repaired TOF patients having PR in the first few decades post-repair [4–9]. Other studies have shown delayed onset of complications within twenty to thirty-seven years post-repair [10, 11]. Follow-up studies, following anatomical correction of TOF repair, report excellent long-term outcomes with a greater than eighty-five percent survival rate up to thirty-six years post-date of surgery at one institution in Munich, Germany [12]. However, data remain limited following the 4th decade of repair [2]. Our case report serves as a rare and delayed presentation of heart failure (HF), 70-year post-TOF repair.

PR with subsequent increased RV pressures leads to HF in patients with surgically amended TOF. PR remains the most common valvular pathology seen before ventricular tachycardia in patients with TOF, and thus interventions aimed at preserving the pulmonic valve are of importance [6–13]. Medical management for patients with TOF undergoing active decompensated heart failure remains the same with preload reduction used to manage right-sided HF while reducing pressure and volume in the RV.

Our patient had preserved left ventricular and right ventricular systolic function. If either had been reduced, it would have been a Class I recommendation for immediate referral to an adult congenital heart disease (ACHD) cardiologist and HF support team per 2018 American Heart Association/American College of Cardiology Guidelines for the Management of Adults with Congenital Heart Disease [13]. By these same guidelines, it is a Class I recommendation for pulmonary valve replacement surgery given that the patient was symptomatic with greater than moderate pulmonic regurgitation. However, given that our patient’s symptoms improved with medical intervention, it was considered a class I recommendation for referral to an ACHD cardiologist with intention of subsequent workup to replace the pulmonic valve.

Our patient had systemic congestion on the right side of his heart, whereas the left side of his heart had systemically low output. Subsequently, a systemic inflammatory response was enacted which led to his presentation involving symptoms of left-sided heart failure when systemic compensatory responses declined.

The timing of PV replacement surgery (rather than intervention) is important, as one must have the replacement surgery before irreversible right ventricular dysfunction takes place. Our patient had serial echos in the outpatient setting according to ACHD guidelines, with only asymptomatic mild pulmonic regurgitation seen prior to this hospitalization. Given the RV dilatation in our patient with greater than moderate pulmonic regurgitation, it is a class I recommendation to plan for PV replacement surgery given the setting of moderate–severe PR [13]. The debate remains regarding surgical versus interventional repair; however, with the increasingly availability of transcatheter valves that fit into the pulmonic outflow tract, the less invasive interventional methods are increasing in popularity.

Routine follow-up testing and intervals for patients with surgically repaired TOF among other congenital heart diseases were also addressed in the 2018 guidelines. Length of time between follow-up appointments, ECG’s, transthoracic echocardiograms (TTE’s), cardiac magnetic resonance/cardiac computerized tomography imaging, and exercise stress testing were addressed in the latest 2018 AHA/ACC Guidelines for ACHD. Our patient would be classified as stage IIc according to the ACHD Anatomy and Physiological classification system for moderate complexity, having prior surgically repaired TOF with significant valvular disease. As such, he will require increased monitoring including a visit with an outpatient ACHD cardiologist every 6–12 months, an ECG and TTE every year, and a CMR, CCT, and exercise stress test every 1–2 years.

Through the above interventions and use of established guidelines, our patient is currently in the 7th decade as his original TOF repair was at the age of 2. To our knowledge, this is currently the oldest survived patient with surgically repaired TOF in the medical literature. The central focus of this case report lies in our deliberate approach to a unique clinical situation. Rather than hastily resorting to invasive valve replacement in response to the patient presentation, we chose a more measured path. We initiated treatment by administering diuretics, a decision rooted in our recognition of the right heart's concomitant volume and, thus, pressure problem. The patient’s initial presentation stemmed from chronic prior TOF surgery, as our patient had excellent follow-up, medical adherence, and was active in pulmonary and cardiac rehabilitation prior to presentation.

- Outcomes past the 4th decade of life for surgically repaired TOF have outcomes which are still under investigation.
- Once greater than moderate PR occurs, it is a class I recommendation for pulmonic valve intervention. However, medical optimization should take place first.
- Following adequate RV load optimization, our patient underwent successful transcatheter pulmonic valve implantation with resolution of symptoms and cessation of diuretic.

## Data Availability

All data generated or analyzed during this case report are included in this published article [and its supplementary information files].

## Abbreviations

ToF: Tetralogy of Fallot
PR: Pulmonary regurgitation
RV: Right ventricle
LV: Left ventricle
